# Thyroid-associated ophthalmopathy: the role of oxidative stress

**DOI:** 10.3389/fendo.2024.1400869

**Published:** 2024-07-11

**Authors:** Chao Ma, Haoyu Li, Shuwen Lu, Xian Li

**Affiliations:** ^1^ Department of Ophthalmology, The First Affiliated Hospital of Zhengzhou University, Zhengzhou, Henan, China; ^2^ Department of Ophthalmology, The Second Xiangya Hospital, Central South University, Changsha, Hunan, China; ^3^ Hunan Clinical Research Centre of Ophthalmic Disease, Changsha, Hunan, China; ^4^ Department of Ophthalmology, The First Affiliated Hospital of Henan University of Chinese Medicine, Zhengzhou, Henan, China; ^5^ Manchester Royal Eye Hospital, Manchester University NHS Foundation Trust, Manchester, United Kingdom; ^6^ Division of Pharmacy and Optometry, School of Health Sciences, Faculty of Biology, Medicine and Health, The University of Manchester, Manchester, United Kingdom

**Keywords:** thyroid-associated ophthalmopathy, oxidative stress, inflammation, therapeutic method, antioxidants

## Abstract

Thyroid-associated ophthalmopathy (TAO) is an autoimmune condition affecting the eyes, characterized by proptosis, extraocular muscle involvement, and in severe cases, vision impairment including diplopia, optic neuropathy, and potential blindness. The exact etiology of TAO remains elusive; however, increased oxidative stress and decreased antioxidant capacity are pivotal in its pathogenesis. Elevated oxidative stress not only directly damages orbital tissues but also influences thyroid function and autoimmune responses, exacerbating tissue destruction. This review explores the role of oxidative stress in TAO, elucidates its mechanisms, and evaluates the efficacy and limitations of antioxidant therapies in managing TAO. The findings aim to enhance understanding of oxidative stress mechanisms in TAO and propose potential antioxidant strategies for future therapeutic development.

## 1 Introduction

Thyroid-associated ophthalmopathy (TAO), also known as Graves’ ophthalmopathy, is an autoimmune condition affecting the eyes, orbit, and surrounding structures (1). TAO presents with a spectrum of ocular manifestations in clinical practice. Symptoms include eyelid retraction, proptosis, forward displacement of the eyeball, periorbital edema, conjunctival injection, and dysfunction of the extraocular muscles (2). Additionally, TAO can impair vision, causing diplopia, reduced visual acuity, and in severe cases, blindness (3). The severity of TAO varies among individuals, significantly impacting quality of life (4). Patients often experience visual disturbances, facial deformities, psychological distress, and increased risk of suicide (5). It is considered the most prevalent orbital disease in adults. While typically associated with Graves’ disease—an autoimmune disorder causing hyperthyroidism—TAO can also occur in patients with normal thyroid function or hypothyroidism, affecting 25% to 58% of those with Graves’ disease (6). Therefore, extensive research is essential to elucidate TAO’s pathogenesis, identify new therapeutic targets, and establish precise treatment strategies.

Despite the intricate interplay of genetic, environmental, and immune factors, the exact mechanisms underlying TAO remain incompletely understood (7). Autoantibodies, such as thyroid stimulating immunoglobulin, likely initiate autoimmune reactions by binding to thyroid stimulating hormone receptors on orbital fibroblasts (8). This triggers production of proinflammatory cytokines, fibroblasts activation, and recruitment of immune cells, resulting in tissue inflammation, swelling, and fibrosis (9). Recent studies have implicated oxidative stress in the pathogenesis of TAO (10). Oxidative stress arises when the production of reactive oxygen species (ROS) exceeds the body’s antioxidant defense mechanisms (11). Patients with TAO exhibit elevated oxidative stress markers, including increased ROS levels, diminished antioxidant capacity, and accumulation of oxidative byproducts in ocular tissues (12, 13). These findings suggest that immune dysfunction and inflammation induced by oxidative stress play significant roles in ocular manifestations associated with TAO.

Oxidative stress contributes to TAO through multiple mechanisms. Firstly, it directly damages ocular tissues, provoking inflammation and immune cell infiltration (14). Secondly, oxidative stress impacts thyroid function and autoimmune responses, indirectly influencing ocular pathology (15). Furthermore, interactions between oxidative stress, inflammation, and autophagy may exacerbate TAO (16). Researchers are actively investigating the role of oxidative stress pathways in TAO to develop novel therapeutic approaches. Currently, antioxidant therapies aimed at neutralizing free radicals have shown promising results in clinical settings (17). Future efforts should focus on elucidating oxidative stress mechanisms in TAO and advancing treatment strategies to enhance patient outcomes and quality of life.

## 2 Oxidative stress in TAO

### 2.1 Basic concept of oxidative stress

Oxidative stress occurs when there is an imbalance between ROS production and the antioxidant defense systems capacity to neutralize them (18). ROS are natural byproducts of cellular metabolism and play essential roles in physiological processes (19). Maintaining ROS within a balanced range is crucial for cellular homeostasis; excess ROS or insufficient antioxidants can lead to oxidative damage and cellular dysfunction (20).

Oxidative stress plays an important role in the pathomechanism of TAO. Oxidative stress is the disruption of the balance between oxygen free radicals and antioxidant systems in the body, leading to cellular damage and tissue injury. Studies have shown that oxidative stress not only directly damages orbital tissues, but also promotes inflammatory responses and fibrosis by activating various signaling pathways (21). Inflammatory response is another important pathological mechanism of TAO. There is a large infiltration of inflammatory cells, such as T cells, B cells, and macrophages, in the orbital tissues of TAO patients. These inflammatory cells further exacerbate orbital tissue damage by secreting various inflammatory factors, such as interleukin-6 (IL-6) and tumor necrosis factor-α (TNF-α) (22). In addition, fibrosis is the final pathological change in the pathologic process of TAO. In TAO, activation and differentiation of fibroblasts are critical steps in the development of fibrosis. Studies have shown that transforming growth factor-β1 (TGF-β1) is an important regulator of TAO fibrosis, which promotes the proliferation of fibroblasts and collagen synthesis by activating the Smad signaling pathway (23). Moreover, oxidative stress and inflammatory responses can also promote fibrosis by activating the TGF-β1 signaling pathway (24). Thus, oxidative stress, inflammatory response, and fibrosis are key mechanisms that are interrelated and mutually reinforcing in the pathologic process of thyroid-related eye disease.

Various lifestyle factors contribute to oxidative stress, potentially triggering the development of TAO, including smoking, environmental exposures, and sleep disturbances (25). Under normal conditions, the body’s antioxidant defenses regulate ROS levels effectively (26). Enzymatic antioxidants such as superoxide dismutase (SOD), catalase, and glutathione peroxidase play pivotal roles in converting ROS like superoxide anions (O^2-^) into less harmful substances such as hydrogen peroxide (H_2_O_2_), which are subsequently detoxified by catalase and glutathione peroxidase (27, 28). Non-enzymatic antioxidants like vitamins E and C, along with glutathione, function as electron donors or scavengers to directly neutralize ROS, thus maintaining cellular redox balance and preventing oxidative damage (29).

**Figure 1:**
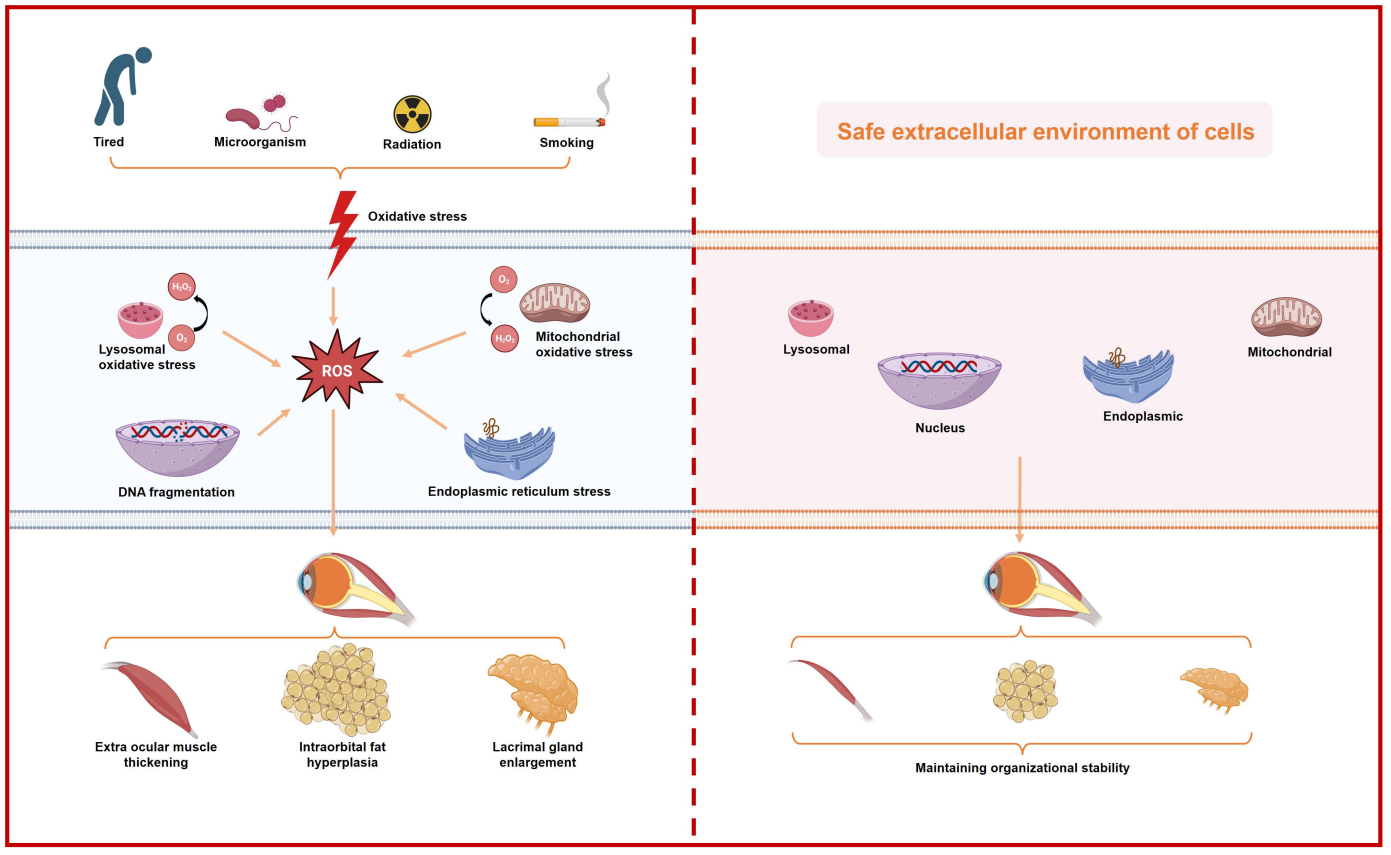
Schematic representation of the increase in intracellular oxidative stress caused by various external factors and its impact on individual organelles, ultimately leading to ocular histopathology in patients with thyroid-associated ophthalmopathy.

Oxidative stress disrupts cellular redox balance, leading to excessive production of oxidants that initiate harmful oxidative reactions. Further investigations reveals that oxidative stress can be categorized as follows (**Figure 1**):

1. Mitochondrial oxidative stress: Mitochondria, crucial for cellular energy production, are primary targets of oxidative stress. Dysfunction in mitochondria can lead to electron leakage in the electron transport chain, resulting in overproduction of ROS such as superoxide anions and hydrogen peroxide (30). These oxidative species can damage mitochondrial DNA, peroxidize membrane lipids, oxidize proteins, and exacerbate mitochondrial dysfunction, creating a detrimental cycle (31).
2. Endoplasmic reticulum oxidative stress: The endoplasmic reticulum (ER), responsible for protein synthesis and folding, is vulnerable to oxidative stress. Disruption in ER function or sudden accumulation of misfolded proteins triggers ER stress responses (32). Oxidative stress in the ER can induce protein oxidation, thiol oxidation, alter calcium ion homeostasis, and perturb cellular signal transduction (33).
3. Lysosomal oxidative stress: Lysosomes, involved in cellular waste degradation, contain enzymes and metal ions that can generate oxidative compounds such as hydrogen peroxide and hydroxyl radicals under oxidative stress conditions (34, 35). Elevated oxidative stress can damage lysosomal membranes and disrupt the acidic internal environment essential for lysosomal function.
4. Nuclear oxidative stress: The nucleus, housing genetic material, is susceptible to oxidative damage. Oxidative stress can induce nuclear DNA damage, alter chromatin structure, interfere with gene transcription, and increase cellular susceptibility to DNA damage (36, 37).
5. Environmental exposure, smoking and late nights: Environmental pollutants, including air pollutants, heavy metals, and chemicals, contribute to oxidative stress by inducing ROS production through oxidation reactions upon exposure via respiratory, digestive, or dermal routes (38). Tobacco smoke, containing harmful chemicals like carbon monoxide and polycyclic aromatic hydrocarbons, directly increases ROS formation upon inhalation, significantly impacting antioxidant defenses and causing cellular damage (39). Sleep disturbances, such as chronic sleep deprivation or circadian rhythm disruption from staying up late, elevate oxidative stress by altering antioxidant enzyme function, impairing immune responses, and promoting inflammation (40).

Oxidative stress in TAO is a well-studied component, implicated in disease initiation and progression (41). Evidence suggests that oxidative stress from immune cells, orbital fibroblasts, and adipocytes infiltrating the orbit contributes to TAO pathogenesis (42). Understanding these mechanisms is critical for developing targeted therapeutic strategies to mitigate oxidative stress and improve outcomes in TAO patients (43).

### 2.2 Relationships between oxidative stress and TAO

The relationship between oxidative stress and TAO involves intricate mechanisms influenced by multiple factors. Imbalance between ROS production and the antioxidant defense system is implicated in TAO pathogenesis (44). Increased ROS production and compromised antioxidant capacity can lead to cellular damage and dysfunction in TAO. Understanding these mechanisms requires exploring oxidative stress at the organelle level and its impact on inflammation and fibrosis, critical components in TAO pathophysiology (45, 46). Oxidative stress perpetuates inflammation, exacerbating tissue damage in a vicious cycle. Additionally, genetic factors and smoking contribute to heightened oxidative stress in orbital tissues of TAO patients (47).

#### 2.2.1 Mitochondrial oxidative stress and TAO

1. Mitochondrial dysfunction: Mitochondria, pivotal for cellular energy production, are implicated in TAO due to their role in thyroid hormone synthesis and metabolism, which consume substantial energy. Impaired mitochondrial function exacerbates hyperthyroidism associated with ocular manifestations in Graves’ disease patients (48, 49).
2. Oxidative stress damage: During oxidative stress, mitochondria generate excessive free radicals and oxidants, disrupting cellular redox balance of cells. This can compromise mitochondrial membrane integrity and DNA, affecting normal ocular tissue function in TAO (50).
3. Inflammatory response: Oxidative stress promotes persistent inflammatory reactions that contribute to ocular tissue damage in TAO (51).

Mitochondrial oxidative stress likely plays a significant role in TAO pathogenesis by influencing thyroid function, damaging ocular tissues, and perpetuating inflammation. Further research on mitochondrial oxidative stress is crucial for understanding the underlying mechanisms of TAO and developing targeted treatments.

#### 2.2.2 Endoplasmic reticulum oxidative stress and TAO

ER oxidative stress occurs when the ER is challenged by various insults, leading to impaired ER function and intracellular oxidative stress (52). Several aspects highlight the connection between TAO and ER oxidative stress:

1. Autoimmune Response Induction: In TAO, thyrotropin receptor antibodies activate thyroid cell receptors, leading to hyperthyroidism and ocular complications. ER oxidative stress increases intracellular free radical generation, triggering inflammation and autoimmune responses (32, 53).
2. Inflammatory response: ER oxidative stress induces the release of inflammatory factors like tumor necrosis factor-α (TNF-α), interleukin-6 (IL-6), which contribute to inflammation, edema, and fibrosis in ocular tissues associated with TAO (54, 55). Furthermore, ER oxidative stress disrupts calcium ion homeostasis, crucial for cell signaling and regulation (56). Calcium imbalance further exacerbates inflammation and fibrosis in TAO (57, 58).
3. Oxidative stress damage: ER oxidative stress increases intracellular oxidative stress, disrupting cellular redox balance. Abnormal protein folding due to ER dysfunction can trigger cell apoptosis and fibrosis, exacerbating disease progression in TAO (59, 60).

Understanding the interplay between ER oxidative stress and TAO requires further basic and clinical research to validate these mechanisms and develop targeted therapeutic strategies.

#### 2.2.3 Lysosomal oxidative stress and TAO

Lysosomes play critical roles in cellular degradation, regulation, and immunity (61). Lysosomal oxidative stress, arising from redox imbalance, contributes to ROS overproduction and inflammation (62). Several factors link lysosomal oxidative stress to TAO:

1. Inflammatory response: Lysosomal oxidative stress triggers inflammatory reactions, involving infiltration and activation of immune cells, exacerbating tissue inflammation and damage in TAO (63). Inflammatory molecules like TNF-α and interleukin-1β are released, contributing to ocular tissue inflammation and fibrosis (64).
2. Apoptosis: Oxidative stress from lysosomal enzymes can induce cell apoptosis, further complicating TAO pathology by promoting tissue damage (65).
3. Immune regulation imbalance: Lysosomal oxidative stress disrupts antigen degradation and presentation, potentially exacerbating autoimmune responses in TAO (66, 67). Lysosomal dysfunction may contribute to adipose tissue hyperplasia and proliferation in orbital tissues associated with TAO (68).
4. Impact on other organelles: Lysosomal oxidative stress can interact with other organelles, such as the ER and mitochondria, affecting cellular signaling and function (34).

Further research is necessary to elucidate the role of lysosomal oxidative stress in thyroid-related eye conditions, confirming and expanding upon existing theoretical frameworks and research findings.

#### 2.2.4 Nuclear oxidative stress and TAO

The nucleus, housing genetic material and regulatory molecules, is vulnerable to oxidative stress-induced damage (69). Nuclear oxidative stress affects TAO through several mechanisms:

1. DNA damage: ROS directly damage DNA in orbital fibroblasts of TAO patients, causing strand breaks, base modifications, and oxidative lesions that disrupt gene expression and DNA repair mechanisms (70).
2. Regulation of transcription factor activity: Oxidative stress influences transcription factor activity, regulating genes involved in inflammation, cell proliferation, apoptosis, and other processes relevant to TAO pathology (71–73).
3. Protein oxidation and modification: Oxidative conditions in the nucleus lead to protein oxidation and modification, altering protein structure and function critical for nuclear processes in TAO (74).
4. Cell cycle and apoptosis regulation: Oxidative stress impacts cell survival and apoptosis pathways, influencing cell cycle progression and apoptotic signaling relevant to TAO progression (75, 76).

Nuclear oxidative stress disrupts cellular functions essential for maintaining ocular tissue integrity in TAO. Further research is essential to comprehensively understand these processes and their implications for therapeutic interventions.

### 2.3 The role of oxidative stress due to environmental exposure, smoking and late nights in the development of TAO

Several factors contribute to the onset of thyroid-associated eye conditions, including environmental exposure, tobacco use, and sleep patterns.

1. Environmental exposure: Air pollutants, heavy metals, chemicals, and radiation constitute environmental exposure. These pollutants enter the body through various pathways, such as the respiratory tract, digestive system, and skin (77). Research links environmental exposure to TAO, where pollutants induce ROS through oxidation reactions, increasing intracellular oxidative stress (78, 79). This oxidative stress activates the immune system, triggering autoimmune reactions that contribute to TAO development (80).
2. Smoking: Smoking is identified as a significant risk factor for TAO development (81). Chemicals in tobacco smoke initiate oxidative reactions, releasing reactive oxygen radicals that elevate oxidative stress levels in cells. Additionally, smoking induces vasoconstriction and inflammatory responses that exacerbate symptoms of thyroid-related eye disease (82).
3. Late nights: Sleep deprivation disrupts the body’s circadian rhythm and adversely affects immune function and metabolism (83). Insufficient sleep leads to increased free radical production, reduced antioxidant enzyme activity, and oxidative stress reactions. Elevated oxidative pressure due to late nights can influence thyroid function and contribute to orbital abnormalities observed in TAO.

Overall, factors like environmental exposure, smoking, and disrupted sleep patterns significantly contribute to the development of thyroid-related eye conditions by increasing free radicals, reducing antioxidants, and disrupting circadian rhythms. Therefore, reducing environmental exposure, quitting smoking and maintaining healthy sleep habits are crucial in preventing and managing thyroid-related eye diseases. Additionally, patients are advised to pursue other effective treatments including medication, surgery, and lifestyle improvements to promote remission and recovery.

### 2.4 Oxidative stress in the process of inflammation and fibrosis in TAO

#### 2.4.1 Oxidative stress and inflammatory pathways in TAO

Oxidative stress plays a pivotal role in the inflammatory pathways associated with TAO. Overproduction of ROS triggers inflammatory responses, prominently involving the nuclear factor kappa-B (NF-κB) pathway, a key regulator of inflammation (84, 85). Activation of NF-κB leads to increased levels of inflammatory cytokines such as IL-1β, IL-6, and TNF-α, contributing to persistent inflammation in TAO (86, 87). Moreover, oxidative stress upregulates adhesion molecules like intercellular adhesion molecule-1 (ICAM-1) and vascular cell adhesion molecule-1 (VCAM-1) on orbital fibroblasts and endothelial cells, facilitating immune cell recruitment and adhesion to inflamed orbital tissue (88, 89). Additionally, oxidative stress activates MAPKs (mitogen-activated protein kinases) including extracellular signal-regulated kinases, c-Jun N-terminal kinases (JNK), and p38 MAPK, which regulate cell growth, differentiation, and inflammation (90, 91). Activation of MAPKs in TAO results in the production of pro-inflammatory mediators and activation of fibroblasts, perpetuating inflammation and fibrosis within the orbit (92).

#### 2.4.2 Oxidative stress and fibrosis of TAO

Oxidative stress also influences the fibrotic processes observed in TAO. ROS stimulate the production of transforming growth factor-β (TGF-β), a major mediator of fibrosis (93, 94). TGF-β promotes transformation of orbital fibroblasts into myofibroblasts, leading to excessive production and accumulation of extracellular matrix components such as collagen and fibronectin (95).

Moreover, oxidative stress disrupts the balance between matrix metalloproteinases (MMPs) and tissue inhibitors of metalloproteinases (TIMPs), critical for collagen turnover and tissue remodeling (96). Excess ROS inhibit MMP activity and expression, impairing collagen degradation and contributing to fibrotic changes observed in TAO, such as increased orbital fat volume and fibrotic tissue expansion (97).

## 3 Therapeutic agents for oxidative stress in TAO and their potential therapeutic targets

### 3.1 Application of antioxidants

**Figure 2:**
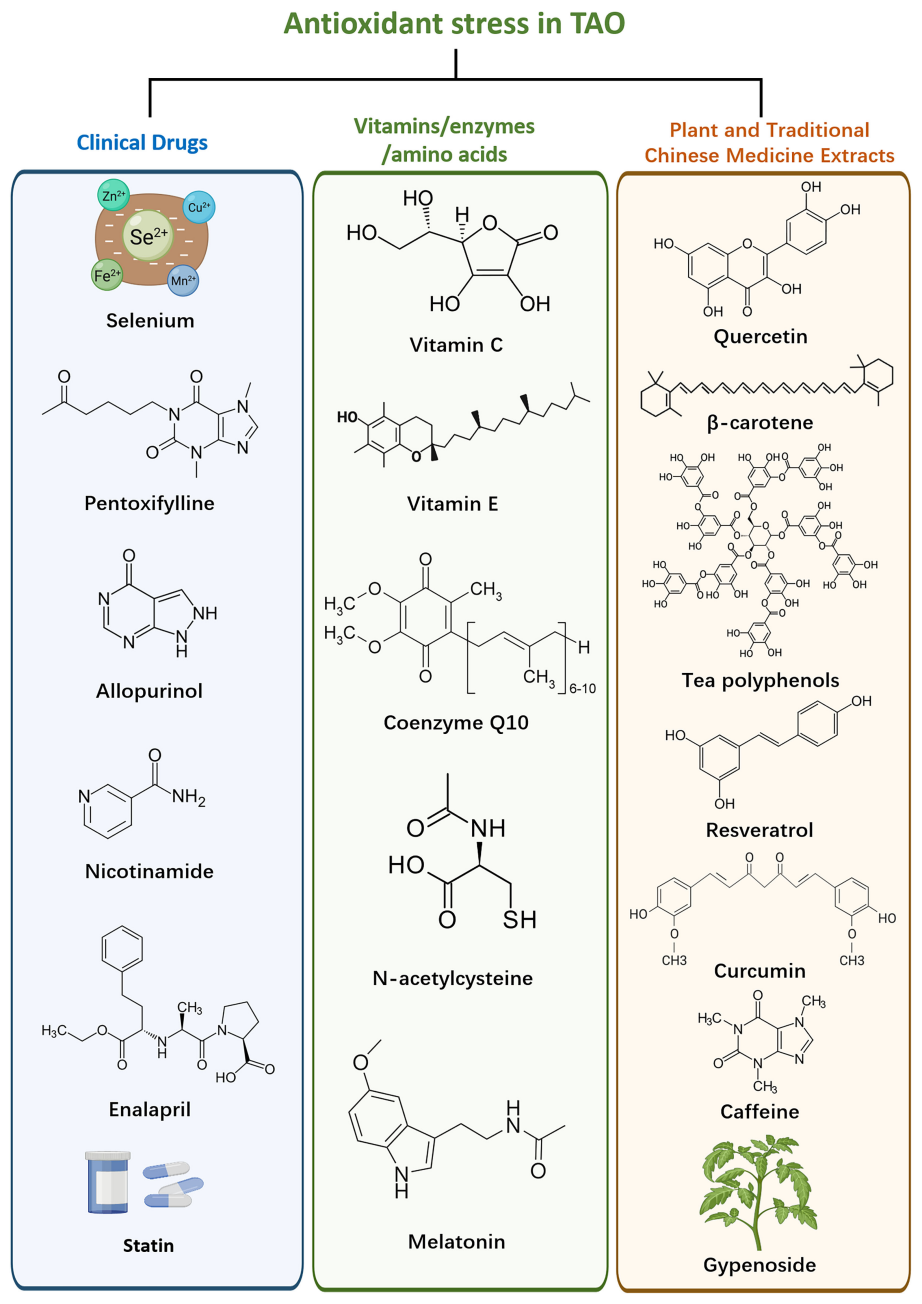
Therapeutic drugs against oxidative stress and their components.

TAO exhibits dysregulation of oxidative stress pathways, which can be targeted to mitigate oxidative damage and restore redox balance (**Figure 2**). One strategy involves the application of antioxidants, which scavenge ROS and reduce oxidative damage in orbital tissues (98).

#### 3.1.1 Clinical application of therapeutic drugs

##### 3.1.1.1 Selenium

Selenium a trace element found in foods like Brazil nuts, tuna, shrimp, meat, eggs, cereals and grains (99), plays a crucial role in thyroid function (100). Selenoproteins, including glutathione peroxidase, thioredoxin reductase, and iodothyronine deiodinase, function as antioxidants regulating redox reactions (101, 102). Selenium also reduces inflammatory cytokine production by preventing NF-B from binding to its gene promoter (103). Consequently, selenium is considered an important supplement in TAO clinical treatment.

##### 3.1.1.2 Pentoxifylline

A xanthine derivative primarily used to treat peripheral vascular diseases (104), pentoxifylline acts as an antioxidant by eliminating excess ROS, thereby reducing inflammation-related damage (105). In TAO patients, pentoxifylline inhibits the proliferation and synthesis of glycosaminoglycans (GAGs) by extraocular myofibroblasts (106). It exerts immunomodulatory effects on cytokine production (107). Suitable for patients with moderate to severe TAO who have normal thyroid function, pentoxifylline can be used without corticosteroids (108). However, further clinical and experimental validation is required to confirm its specific dosage and effectiveness.

##### 3.1.1.3 Allopurinol and Nicotinamide

*In vitro* studies show that allopurinol and nicotinamide prevent superoxide-induced growth of orbital fibroblasts in TAO patients (109). Nicotinamide reduces the expression of human leukocyte antigen-DR and ICAM-1 stimulated by cytokines in orbital fibroblasts during the active phase of TAO (110). Additionally, it suppresses the growth of orbital fibroblasts and reduces HSP72 expression induced by superoxide (111). A clinical trial found that 82% of patients with mild to moderately severe, recently diagnosed active TAO experienced a decrease in the severity of orbital lesions after three months of oral allopurinol and nicotinamide treatment, compared to 27% in the control group (112). Further clinical and experimental evidence is necessary to confirm the efficacy and safety of nicotinamide and allopurinol in TAO treatment.

##### 3.1.1.4 Enalapril

This ACE inhibitor, commonly used for hypertension, protects tissues from oxidative damage by enhancing antioxidant defenses and decreasing ROS levels (113). Enalapril supports glutathione-dependent antioxidant protection (114). *In vitro* studies demonstrated its antiproliferative and anti-hyaluronic acid (HA) secretion effects in both TAO patient groups and non-TAO control fibroblasts (115). A clinical trial with 12 individuals with mild TAO showed improvements in eye bulging, CAS scores, eyelid retraction, and overall well-being compared to the control group (116). Enalapril’s positive impact on TAO is likely due to its antioxidant properties, inhibition of orbital fibroblast growth, and reduction of HA accumulation. However, the limited sample size necessitates additional research to confirm its efficacy in moderate and severe TAO cases.

##### 3.1.1.5 Statins

Statins, inhibitors of 3-hydroxy-3-methylglutaryl coenzyme A reductase, are widely used to treat hypercholesterolemia (117). They reduce oxidative stress by lowering blood lipids, eliminating free radicals, and inhibiting vascularity (118). Evidence suggests that statins may reduce the risk of thyroid-related adverse events in patients with thyroid dysfunction (119, 120). Clinical trials have demonstrated that oral atorvastatin can improve the quality of life and reduce eye lesions in active TAO patients with hypercholesterolemia compared to intravenous corticosteroids (121). Statins may prevent the onset and progression of TAO in newly diagnosed thyroid dysfunction patients, warranting further clinical trials to investigate their prophylactic potential (122). The therapeutic feasibility of statins may be explained by the potential link between TAO and increased free fatty acid load, which leads to ROS and proinflammatory cytokine release implicated in TAO pathogenesis (123).

#### 3.1.2 Vitamins, enzymes and amino acids

##### 3.1.2.1 Vitamin C

Ascorbic acid, essential for wound healing and collagen synthesis, act as a reducing agent in redox reactions (124). Vitamin E: This fat-soluble antioxidant scavenges ROS in the body (125). Ubiquinone (Coenzyme Q10): Essential for aerobic respiration, it prevents free radicals interaction with antioxidant molecules in the body (126). N-acetylcysteine: A precursor to glutathione, it helps decrease free radical generation during oxidative stress (127). Melatonin: Released by the pineal gland to regulate circadian rhythm melatonin scavenges free radicals and enhances antioxidant activity (128).

N-acetylcysteine and vitamin C inhibit H_2_O_2_-induced fibroblast proliferation and reduce TGF-β1, IL-1β, and superoxide anion expression in TAO fibroblasts (129). Studies on primary cultures of orbital fibroblasts from TAO patients have shown that vitamin C, N-acetylcysteine and melatonin reduce H_2_O_2_-stimulated glutathione disulphide (GSSG) release, potentially benefiting orbital lesion treatment (130). Additionally, vitamin C and E supplementation improved clinical symptoms and clinical scores in TAO patients by reducing oxidative stress markers (131). These antioxidants inhibit TAO fibroblast proliferation, with N-acetylcysteine and melatonin selectively blocking IFN-γ release (132). They also reduce HA and IL-1β levels in orbital fibroblasts of TAO patients and normal controls (133). Despite promising results, the complexity of the body’s circulatory metabolism necessitates in-depth clinical studies to evaluate each antioxidant’s therapeutic efficacy.

#### 3.1.3 Plant extracts

Natural plant extracts with antioxidant properties, such as polyphenols, curcumin, resveratrol and quercetin, show potential benefits in reducing oxidative stress and inflammation in TAO (134). The compounds act as ROS scavengers, inhibit ROS-generating enzymes, and activate the endogenous antioxidant system (135). The effectiveness of these compounds in reducing oxidative stress and improving tissue damage in TAO models has been demonstrated in preclinical studies (136).

##### 3.1.3.1 Quercetin

A polyphenolic flavonoid found in fruits, vegetables, red wine and tea, quercetin has antioxidant, anti-inflammatory, and antiproliferative properties (137, 138). In TAO, quercetin regulates inflammatory pathways, extracellular matrix molecule accumulation, and adipose tissue growth in primary cultured orbital fibroblasts. It suppresses the production of ICAM-1, IL-6, IL-8, and cyclooxygenase 2 mRNA, and prevents IL-1β-induced elevations of these molecules (139). Therefore, quercetin’s effects may alleviate clinical symptoms of orbital lesions in TAO patients.

##### 3.1.3.2 β-carotene

A carotenoid widely found in plants; β-carotene is an antioxidant with neuroprotective effects (140, 141). It reduces H_2_O_2_-induced GSSG production, IL-1β levels, and cell proliferation in TAO orbital fibroblasts (142). However, overconsumption can cause carotenosis, an orange pigmentation of the skin, and may increase lung cancer risk in smokers (143, 144). Further investigation is needed to evaluate β-carotene’s long-term therapeutic effects and safety in TAO.

##### 3.1.3.3 Tea polyphenols (TP)

Derived from tea leaves, TP compounds, primarily catechins, provide antioxidant and anti-inflammatory benefits (145, 146). Epigallocatechin-3-gallate, the main component of green tea extract, reduces IL-1β and exerts anti-inflammatory effects in TAO orbital fibroblasts (147). TP also modulates NF-κB/NLRP3 pathway, reducing IL-6, IL-1β, and MCP-1 expression in LPS-induced TAO orbital fibroblasts (148). However, TP treatment for TAO remains experimental, requiring further research to confirm its effectiveness and safety.

##### 3.1.3.4 Resveratrol

A non-flavonoida phenolic compound from red grapes, berries, and peanuts, resveratrol has antioxidant, anticancer, anti-inflammatory, anti-apoptosis, and free radical scavenging properties (149). In TAO, it reduces ROS generated by cigarette smoke extract or H_2_O_2_ and during adipogenesis in primary cultured orbital fibroblasts (150). Although promising in animal studies, there is no reliable evidence of resveratrol’s benefits for human health. Further research is necessary to understand its mechanism and therapeutic role in TAO.

##### 3.1.3.5 Curcumin

A plant polyphenol from turmeric with demonstrated antioxidant and anti-inflammatory activity *in vitro*, *in vivo*, and in humans (151, 152). *In vitro* studies show curcumin inhibits cell proliferation, adipogenesis, ROS production, and IL-1β-induced inflammatory responses in TAO patients’ orbital fibroblasts (153). While its anticancer effects are well-documented, curcumin’s poor human absorption and bioavailability limit its clinical use (154, 155). Additional studies on efficacy and safety are required.

##### 3.1.3.6 Caffeine (1,3,7-trimethylxanthine)

A plant alkaloid, which is commonly found in beverages such as coffee, tea, chocolate, and cola (156). Numerous studies have identified it as an oxygen radical scavenger, demonstrating both *in vitro* and *in vivo* antioxidant activity in animals and humans, and suggesting it may reduce inflammatory responses (157, 158). In the context of treating TAO, caffeine appears to inhibit orbital changes by reducing ROS production in response to cellular oxidative stress and exhibiting anti-adipogenic effects (159). However, excessive caffeine consumption can lead to adverse effects, including addiction, nervousness, irritability, anxiety, tremors, muscle twitching, insomnia, and heart palpitations (160). Therefore, determining the appropriate dosage to alleviate TAO-related orbital lesions without inducing adverse effects is an important area for future research.

##### 3.1.3.7 Gynostemma pentaphyllum

Gynostemma pentaphyllum is a herbaceous climbing plant, and its derivatives, particularly gypenosides (Gyps), have long been used as safe and effective natural remedies for various diseases. Gyps exhibit different oral bioavailability values and a limited ability to cross the blood-brain barrier (161). Gyps have been shown to reduce oxidative stress induced by H_2_O_2_ in orbital fibroblasts from TAO patients, thereby decreasing cellular autophagy and apoptosis, and consequently minimizing orbital tissue damage (162). Additionally, Gyps inhibit the activation of the Toll-like receptor 4/NF-κB signaling pathway and the TGF-β1/SMAD2/SMAD4 signaling pathway in orbital fibroblasts, exerting anti-inflammatory and antioxidant effects (163). While Gyps have demonstrated protective effects on orbital fibroblasts in experimental settings, improving their bioavailability remains a key focus for their clinical application.

### 3.2 Regulation of organismal oxidative stress

**Figure 3:**
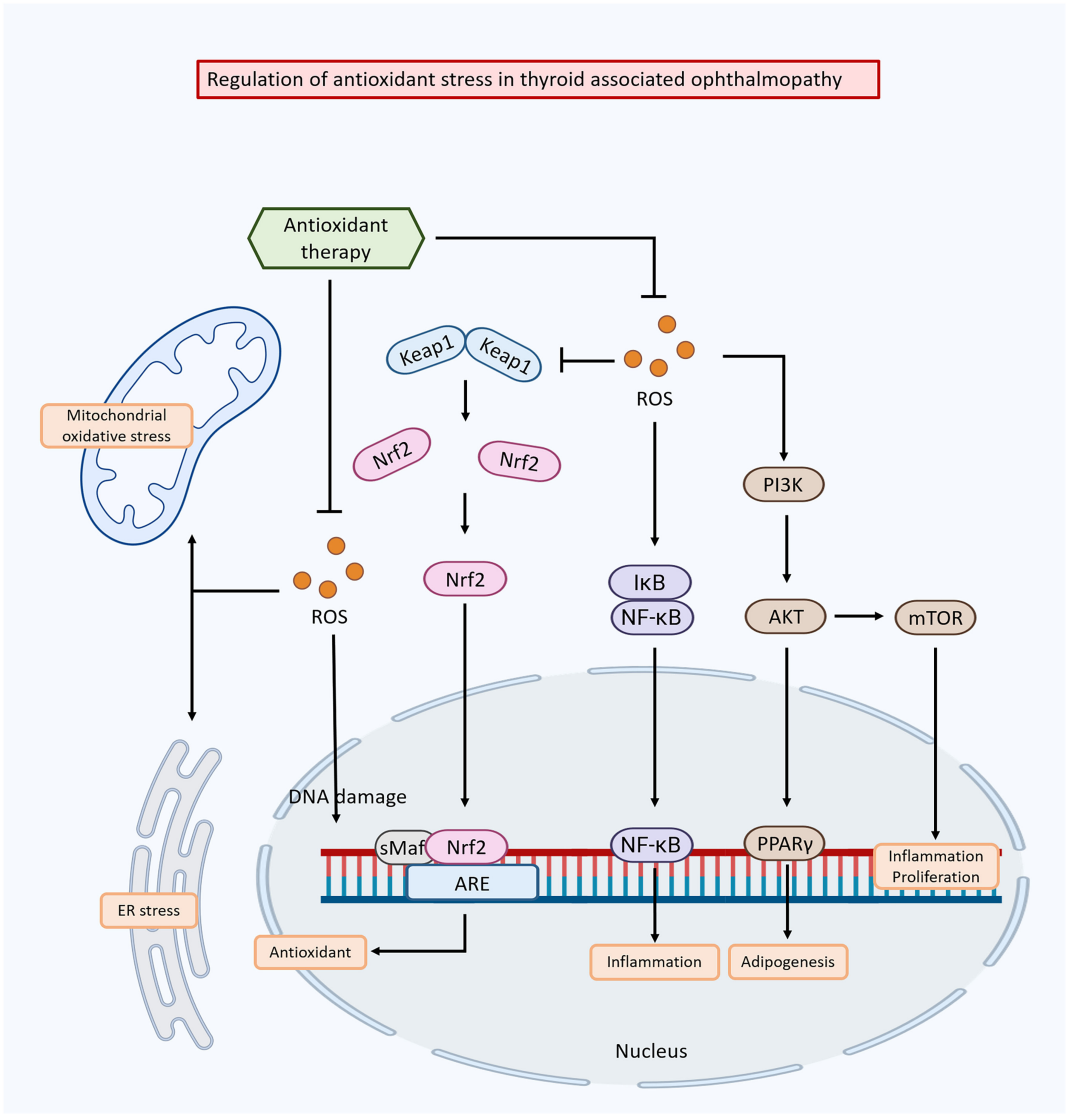
Mechanisms of oxidative stress-induced damage in thyroid-associated ophthalmopathy and the modulation of these effects by anti-oxidative stress drugs.

Dysregulation of oxidative stress pathways plays a critical role in the pathogenesis of TAO (**Figure 3**). Targeting these pathways has emerged as a potential therapeutic strategy to mitigate oxidative damage, reduce inflammation, and improve clinical outcomes in TAO. One approach involves regulating enzyme systems responsible for ROS production, such as NADPH oxidase and xanthine oxidase. These enzymes produce ROS as by-products of their normal physiological function, and their upregulation or overactivation can lead to oxidative stress in TAO (164). Inhibiting or modulating these enzymes has been shown to reduces ROS production and subsequent oxidative damage (165).

Another strategy is to enhance the antioxidant defense system to clear ROS and restore redox balance. This can be achieved by activating endogenous antioxidant enzymes such as SOD, catalase, and glutathione peroxidase, which neutralize ROS and prevent oxidative damage (166, 167). The nuclear factor erythroid 2-related factor 2 (Nrf2) pathway is a key transcription factor that regulates the expression of antioxidant-responsive genes, playing a crucial role in this process (168).

The Nrf2 pathway induces broad-spectrum antioxidant responses and mitigates damage from inflammatory factors (169). Under normal conditions, Nrf2 is sequestered in the cytoplasm by its inhibitor protein, Kelch-like ECH-associated protein 1 (Keap1). When oxidative stress occurs, Nrf2 dissociates from Keap1 and translocates to the nucleus, where it binds to antioxidant response elements (AREs) in the promoter regions of specific genes, initiating their transcription process (170). The Keap1/Nrf2 system significant contributes to maintaining redox and metabolic homeostasis, demonstrating positive effects in various disease models, including inflammatory, autoimmune, metabolic, and neurodegenerative conditions (171).

Activation of the Nrf2 pathway shows promising outcomes in TAO. Nrf2 activation attenuates oxidative stress in orbital fibroblasts, reduces inflammation, and ameliorates orbital tissue damage in TAO patients (172). Additionally, Nrf2 activation has been associated with reduced fat aggregation in TAO and improved clinical outcomes (173). Conversely, blocking Nrf2 transcriptional activity in leukemia cells leads to ROS accumulation and cell death via apoptosis (174). Vitamin C, a well-known antioxidant, enhances the sensitivity of cancer cells resistant to imatinib by disrupting the Nrf2/ARE complex, resulting in decreased expression of the catalytic subunit of glutamate-cysteine ligase and lower GSH levels (175).

The regulation of Nrf2 varies across different diseases, and it could be beneficial in identifying biomarkers and potential drug targets for thyroid diseases during TAO treatment. Therefore, key areas for future investigation include exploring the functions and pathways of Nrf2-triggered antioxidants in TAO and examining the potential effectiveness of Nrf2 regulation in modulating autoimmune responses in TAO. Modulation of oxidative stress pathways as therapeutic targets for TAO offers a promising avenue for new interventions. Further studies are needed to elucidate the specific molecular mechanisms of oxidative stress in TAO and to assess the safety and efficacy of therapeutic agents targeting these pathways. Addressing oxidative stress may help attenuate tissue damage, reduce inflammation, and improve clinical outcomes in TAO.

## 4 Summarization and prospect

Oxidative stress plays a significant role in the pathogenesis of TAO. An imbalance between ROS production and antioxidant defenses lead to oxidative stress, resulting in cellular damage and dysfunction. Multiple factors contribute to the relationship between oxidative stress and TAO, including the activation of inflammatory pathways, genetic and environmental factors, and dysregulation of antioxidant defense mechanisms. The relationship between oxidative stress and TAO is complex and multifaceted. ROS in TAO are produced by infiltrating immune cells, orbital fibroblasts and adipocytes, leading to tissue damage and inflammation. Elevated oxidative stress activates inflammatory pathways, perpetuating the cycle of tissue damage and inflammation in TAO.

Addressing oxidative stress in TAO is critical for developing effective therapeutic strategies. Antioxidants, such as vitamins C and E, and activation of endogenous antioxidant defense mechanisms have shown promise in reducing oxidative stress and inflammation in TAO. Further studies are needed to fully understand the complex mechanisms of oxidative stress and its role in the pathogenesis of TAO. Future research could focus on several areas to enhance our knowledge and improve therapeutic strategies for TAO:

1. Exploring the interactions between oxidative stress and other key pathways: Investigating the interactions between oxidative stress and the inflammation, fibrosis, and immune dysregulation that characterize TAO could lead to a more comprehensive understanding of the disease. Elucidating these interactions could reveal underlying mechanisms and aid in developing targeted therapies that address multiple aspects of the disease.
2. Identification of new therapeutic targets: Identifying new targets within the oxidative stress pathway could provide novel therapeutic opportunities. This could involve targeting specific enzymes or molecules involved in ROS production, such as NADPH oxidase or xanthine oxidase.
3. Modulation of antioxidant defense mechanisms: Exploring ways to enhance endogenous protection against oxidative stress, such as through the Nrf2 pathway, could be beneficial. Understanding the regulation and activation of Nrf2 in TAO could inform new therapeutic strategies.

In conclusion, future research efforts should focus on unraveling the complex interactions between oxidative stress and other key pathways in TAO, identifying new therapeutic targets, and conducting robust clinical studies to validate the effectiveness of antioxidant interventions. By improving our understanding and developing targeted therapeutic approaches, we can endeavor to enhance management strategies and improve the quality of life for TAO patients.

## Author contributions

CM: Funding acquisition, Resources, Supervision, Writing – original draft, Writing – review & editing. HL: Data curation, Writing – review & editing. SL: Methodology, Writing – review & editing. XL: Supervision, Writing – original draft.
